# Comparison of O-specific polysaccharide responses in patients following infection with *Vibrio cholerae* O139 versus vaccination with a bivalent (O1/O139) oral killed cholera vaccine in Bangladesh

**DOI:** 10.1128/msphere.00255-23

**Published:** 2023-08-30

**Authors:** M. Hasanul Kaisar, Meagan Kelly, Mohammad Kamruzzaman, Taufiqur R. Bhuiyan, Fahima Chowdhury, Ashraful Islam Khan, Regina C. LaRocque, Stephen B. Calderwood, Jason B. Harris, Richelle C. Charles, Alžbeta Čížová, Jana Mečárová, Jana Korcová, Slavomír Bystrický, Pavol Kováč, Peng Xu, Firdausi Qadri, Edward T. Ryan

**Affiliations:** 1 International Centre for Diarrhoeal Disease Research Bangladesh (icddr,b), Dhaka, Bangladesh; 2 Division of Infectious Diseases, Massachusetts General Hospital, Boston, Massachusetts, USA; 3 Department of Medicine, Harvard Medical School, Boston, Massachusetts, USA; 4 Department of Pediatrics, Harvard Medical School, Boston, Massachusetts, USA; 5 Division of Global Health, MassGeneral Hospital for Children, Boston, Massachusetts, USA; 6 Department of Immunology and Infectious Diseases, Harvard T.H. Chan School of Public Health, Boston, Massachusetts, USA; 7 Institute of Chemistry, Slovak Academy of Sciences, Bratislava, Slovakia; 8 Department of Chemical Theory of Drugs, Faculty of Pharmacy, Comenius University in Bratislava, Bratislava, Slovakia; 9 Laboratory of Bioorganic Chemistry (LBC), National Institute of Diabetes, Digestive and Kidney Diseases (NIDDK), National Institutes of Health, Bethesda, Maryland, USA; University of Florida, Gainesville, Florida, USA

**Keywords:** O-specific polysaccharide, *Vibrio cholerae *O139, cholera, immune response, oral cholera vaccine

## Abstract

Cholera caused by *Vibrio cholerae* O139 emerged in the early 1990s and spread rapidly to 11 Asian countries before receding for unclear reasons. Protection against cholera is serogroup-specific, which is defined by the O-specific polysaccharide (OSP) component of lipopolysaccharide (LPS). *V. cholerae* O139 also expresses the OSP-capsule. We, therefore, assessed antibody responses targeting *V. cholerae* O139 OSP, LPS, capsule, and vibriocidal responses in patients in Bangladesh with cholera caused by *V. cholerae* O139. We compared these responses to those of age-gender-blood group-matched recipients of the bivalent oral cholera vaccine (OCV O1/O139). We found prominent OSP, LPS, and vibriocidal responses in patients, with a high correlation between these responses. OSP responses primarily targeted the terminal tetrasaccharide of OSP. Vaccinees developed OSP, LPS, and vibriocidal antibody responses, but of significantly lower magnitude and responder frequency (RF) than matched patients. We separately analyzed responses in pediatric vaccinees born after *V. cholerae* O139 had receded in Bangladesh. We found that OSP responses were boosted in children who had previously received a single dose of bivalent OCV 3 yr previously but not in vaccinated immunologically naïve children. Our results suggest that OSP-specific responses occur during cholera caused by *V. cholerae* O139 despite the presence of capsules, that vaccination with bivalent OCV is poorly immunogenic in the short term in immunologically naïve individuals, but that OSP-specific immune responses can be primed by previous exposure, although whether such responses can protect against O139 cholera is uncertain.

**IMPORTANCE:**

Cholera is a severe dehydrating illness in humans caused by *Vibrio cholerae* serogroups O1 or O139. Protection against cholera is serogroup-specific, which is defined by the O-specific polysaccharide (OSP) of *V. cholerae* LPS. Yet, little is known about immunity to O139 OSP. In this study, we assessed immune responses targeting OSP in patients from an endemic region with cholera caused by *V. cholerae* O139. We compared these responses to those of the age-gender-blood group-matched recipients of the bivalent oral cholera vaccine. Our results suggest that OSP-specific responses occur during cholera caused by *V. cholerae* O139 and that the OSP responses primarily target the terminal tetrasaccharide of OSP. Our results further suggest that vaccination with the bivalent vaccine is poorly immunogenic in the short term for inducing O139-specific OSP responses in immunologically naïve individuals, but OSP-specific immune responses can be primed by previous exposure or vaccination.

## INTRODUCTION

Epidemic cholera has been associated with two serogroups of *Vibrio cholerae*: O1 and O139 (1). Cholera caused by *V. cholerae* O139 emerged in the early 1990s and spread rapidly to 11 Asian countries before receding for unclear reasons (2 – 5). Although *V. cholerae* O139 has not been associated with large outbreaks for over a decade, it is still reported sporadically in Bangladesh (6 – 8), India (9), and China (10), indicating that it could re-emerge as a public health concern (11). Protection against cholera is serogroup-specific, and serogroup-specificity is defined by the O-specific polysaccharide (OSP) component of bacterial lipopolysaccharide (LPS) (12 – 14). Although the oligosaccharide cores of *V. cholerae* O1 and O139 are the same (15), the OSP of *V. cholerae* O139 is distinct from that of *V. cholerae* O1. The latter is comprised of repetitive *N*-(3-deoxy-L-*glycero*-tetronyl)-D-perosamines (16), while the former is a single hexasaccharide (17 – 20). Furthermore, *V. cholerae* O139 expresses a capsule comprised of a polymer of the OSP-hexasaccharide (15, 18 – 21), while *V. cholerae* O1 is unencapsulated. We have previously characterized the immune response to O139 OSP and its fragments using pooled human patient serum samples and found that the terminal tetrasaccharide of the OSP-hexasaccharide is particularly immunogenic (22). Despite the importance of OSP-specific responses in mediating protection against cholera caused by *V. cholerae* O1 (23), little is known about OSP-specific responses in individual patients infected with *V. cholerae* O139 and in vaccine recipients of bivalent (O1/O139) oral killed cholera vaccine (OCV) currently included in the global stockpile of cholera vaccine and used in cholera control programs. Here, we characterize O139 OSP-specific antibody responses in individual patients with cholera caused by *V. cholerae* O139 during an outbreak in 2002 in Bangladesh and compare these responses to those induced by OCV in the age-gender-blood group matched participants in a vaccine study in Bangladesh in 2017.

## MATERIALS AND METHODS

### Study design and specimen collection

This study used samples from 23 diarrheal patients admitted at the icddr,b hospital, Dhaka, Bangladesh, during a cholera epidemic in 2002 (24). All patients had *V. cholerae* O139 isolated from stool and had moderate to severe dehydration from diarrhea. In addition, this study included samples from 23 cholera vaccinees from an OCV clinical trial conducted in Mirpur, Dhaka in 2017, who were matched to patients by age, sex, and blood group (O versus non-O) (25). All vaccinees received two doses of bivalent (O1/O139) oral killed cholera vaccine (Shanchol; Sanofi-Shanta Biotech, India) separated by 2 weeks. Samples from 20 young children vaccinees <5 yr of age who participated in a 2014 vaccine trial, and the 2017 vaccine trial described above were also included in this analysis. Among these 20 vaccinees, 10 had previously received a single dose of bivalent (O1/O139) vaccine in 2014, while the other 10 had received a placebo in the 2014 single dose trial of oral cholera vaccine conducted in Dhaka (26, 27). These 20 children were subsequently included in the 2017 two-dose evaluation of the bivalent (O1/O139) vaccine in Bangladesh, affording us the ability to assess whether previously confirmed exposure to bivalent (O1/O139) vaccine could facilitate boosting of anti-O139 antibody responses during subsequent vaccination (25). The demographic characteristics of all the participants included in the study are provided in Tables 1 and 2.

**TABLE 1:** Demographic characteristics of the cholera adult patients (2002) and vaccinees (2017)

Characteristics	Patients	Vaccinees	*P* values
Sex			
Male, number (%)	11 (47.8)	12 (52.2)	1.00
Female, number (%)	12 (52.2)	11 (47.8)	
Median age, year (25th, 75th percentiles)	30.8 (22.8, 35.8)	29.5 (21.5, 37)	0.9125
Blood group			
O, number (%)	10 (43.5)	7 (30.4)	1.00
Non-O, number (%)	13 (56.5)	16 (69.6)	

**TABLE 2:** Demographic characteristics of recipients under 5 yr of age of two doses O1/O139 vaccine in 2017, who had previously received a placebo or a single dose of O1/O139 vaccine in 2014

Characteristics	Vaccinees receiving prior dose	Vaccinees receiving no prior dose	
Sex			
Male, number (%)	5 (50)	5 (50)	
Female, number (%)	5 (50)	5 (50)	
Median age, year (25th, 75th percentile)	4 (4, 4)	4 (4, 4)	
Blood group			
O, number (%)	5 (50)	5 (50)	
Non-O, number (%)	5 (50)	5 (50)	

All samples were collected following written informed consent and/or assent, including permission for subsequent analysis. The 2014 vaccine study was approved by the Institutional Review Board of icddr,b, and the 2017 vaccine study was approved by the Institutional Review Boards of both icddr,b and the International Vaccine Institute (IVI). The collection and analysis of clinical samples from patients with *V. cholerae* O139 and use of all samples for this current analysis were approved by the Institutional Review Boards of icddr,b and the Massachusetts General Hospital. Venous blood was drawn from patients 2 days after hospitalization and clinical stabilization and then on days 7 and 21; venous blood was drawn from vaccinees on days 0, 14, and 28. Vaccinees in the 2017 cohort received bivalent (O1/O139) vaccine on days 0 and 14. All blood samples were collected and separated to obtain serum or plasma, which were subsequently stored at −80°C until further use in immunological assays.

### Preparation of lipopolysaccharide, O-specific polysaccharide, its terminal tetrasaccharide, and capsule from *V. cholerae* O139

LPS and OSP-core from *V. cholerae* O139 strain CIRS245, synthetic terminal tetrasaccharide, and OSP and its terminal tetrasaccharide fragment conjugated to bovine serum albumin (BSA; for immunologic assays) were generated as previously described (17, 22). Capsule was purified from *V. cholerae* O139 strain CIRS245 via phenol-water extraction. Briefly, capsule was obtained by repeated (twice) extraction of wet biomass of CIRS245 with 1:1 phenol (90%)—water (v/v) at 68°C for 30 min. The extract was dialyzed against tap water for 24 h followed by deionized water for 2–4 h using a MWCO 12,000 membrane (Serva, Germany). Separation of capsule was achieved by ultracentrifugation of retentate (5 h; 136,057 × *g*; 4°C). Supernatant containing capsule and nucleic acids was freeze-dried, then suspended in 50 mM Tris-HCl (pH 7.5) with 1 mM CaCl_2_ and 2 mM MgCl_2_ (40 mL/10 mg of material) and incubated with 50 µg DNase from bovine pancreas (Sigma, Germany) along with 50 µg RNase A from bovine pancreas (Sigma, Germany) overnight at 37°C, followed by 100 µg Proteinase K from *Tritirachium album* (Sigma, Germany) for 4 h at 37°C. Enzymes were inactivated by heating at 80°C for 1 h, and the material was dialyzed through a MWCO 3500 membrane (Serva, Germany). The capsule was further purified by repeated (three times) ultrafiltration using 100,000 MWCO Amicon Ultra 4 mL filters (Merck, Germany) followed by size exclusion chromatography on a Bio-Gel P30 column (Bio-Rad Laboratories, USA) eluted with water. The first eluting double peak detected in the refractive index chromatogram corresponding to the capsule was concentrated and freeze-dried. Absence of nucleic acids and proteins was verified spectrophotometrically using Shimadzu UVmini-1240 UV-VIS spectrophotometer by measuring absorbance at 256 nm and 280 nm, respectively. In order to remove residual traces of LPS detected on SDS-PAGE using Bio-Rad Mini-PROTEAN Cell system, an extra step of ultracentrifugation was performed as described above.

### Antibody responses to O-specific polysaccharide, terminal tetrasaccharide, lipopolysaccharide, and capsule of *V. cholerae* O139

Antibody responses in plasma or serum against O-specific polysaccharide, terminal tetrasaccharide, lipopolysaccharide, and capsule of *V. cholerae* O139 were assessed using ELISA as previously described (28, 29). Briefly, 96-well polystyrene plates (NuncF, Denmark) were coated per well with 100 µL of the specific antigen (OSP:BSA, LPS, tetrasaccharide:BSA, capsule) and then blocked in 1% BSA (Sigma, St. Louis, MO) in phosphate-buffered saline (PBS). All antigens were coated in 50 mM carbonate buffer (pH 9.6) at a concentration of 1 µg/mL, except for LPS, which was coated at 2.5 µg/mL. Each serum or plasma sample was diluted 1:50 in 0.1% BSA in PBS-0.05% Tween and added in triplicate (100 µL per well) to the coated plate, followed by incubation for 90 min at 37°C. Antigen-specific IgA, IgG, and IgM antibodies in samples were detected using horseradish peroxidase-conjugated rabbit anti-human antibodies of the relevant isotype (1:1,000 dilution; Jackson ImmunoResearch, West Grove, PA) as secondary antibodies. Following further incubation for 90 min, bound secondary antibodies were detected using *o*-phenylene diamine (Sigma, St. Louis, MO) in 0.1 M sodium citrate buffer (pH 4.5) and 0.012% hydrogen peroxide. Optical density was measured at 450 nm for 5 min at 14 s intervals, and the rate of change in optical density was expressed as milli-absorbance units per minute. The mean absorbance value for each sample was normalized to ELISA units by dividing the value for the sample against that of a pooled convalescent-phase sera standard. A responder was defined as any patient or vaccinee who had a ≥1.5-fold increase in ELISA units at the assessed time compared to day 2 or day 0, respectively.

### Vibriocidal antibody assay

Vibriocidal antibody titers were measured using serum or plasma from patients and vaccinees as previously described with a less encapsulated strain of *V. cholerae* O139 CIRS134B as the target organism (24). The vibriocidal titer was defined as the reciprocal of the highest dilution of the serum or plasma, resulting in a greater than 50% reduction in optical density compared to that of control wells containing no serum or plasma. A responder was defined as any patient or vaccinee who had a ≥fourfold increase in vibriocidal titer at the assessed time point compared to day 2 or day 0, depending on the cohort.

### Statistical analyses

Differences in demographic characteristics between the two groups were tested using the Wilcoxon signed-rank test or the Fischer’s exact test, as appropriate. Differences in immune responses within groups were assessed using the Wilcoxon signed-rank test, while differences between groups were assessed using the Mann–Whitney *U* test. Linear relationships between immune responses were evaluated using the Spearman rank correlation coefficient. All reported *P* values were two-tailed, with a cutoff of *P* ≤ 0.05 considered a threshold for statistical significance. All statistical analyses were performed using GraphPad Prism 7 (GraphPad Software, Inc.).

## RESULTS

### Plasma antibody responses to OSP, tetrasaccharide, LPS, and capsule

**Fig 1:**
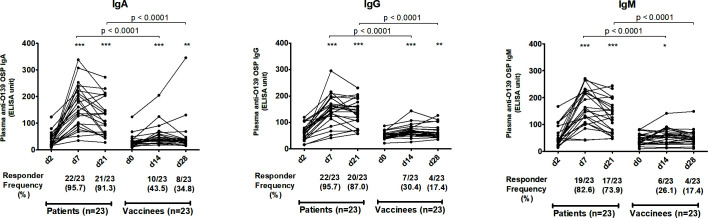
Plasma antibody responses against *V. cholerae* O139 O-specific polysaccharide in naturally infected patients and bivalent O1/O139 vaccinees. *P* values represent statistical differences in the mean between patients and vaccinees. Asterisks represent statistically significant differences in immune responses within patients or vaccinees compared to baseline (***P ≤ 0.001, **P ≤ 0.01, and *P ≤ 0.05). Responder frequencies are shown in parentheses below the X-axes (see text).

**Fig 2:**
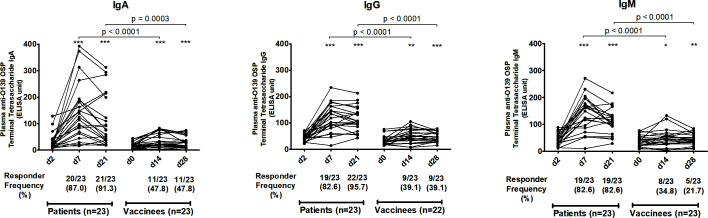
Plasma antibody responses against *V. cholerae* O139 terminal tetrasaccharide of O-specific polysaccharide in naturally infected patients and bivalent O1/O139 vaccinees. *P* values represent statistical differences in the mean between patients and vaccinees. Asterisks represent statistically significant differences in immune responses within patients or vaccinees compared to baseline (***P ≤ 0.001, **P ≤ 0.01, and *P ≤ 0.05). Responder frequencies are shown in parentheses below the X-axes (see text).

**Fig 3:**
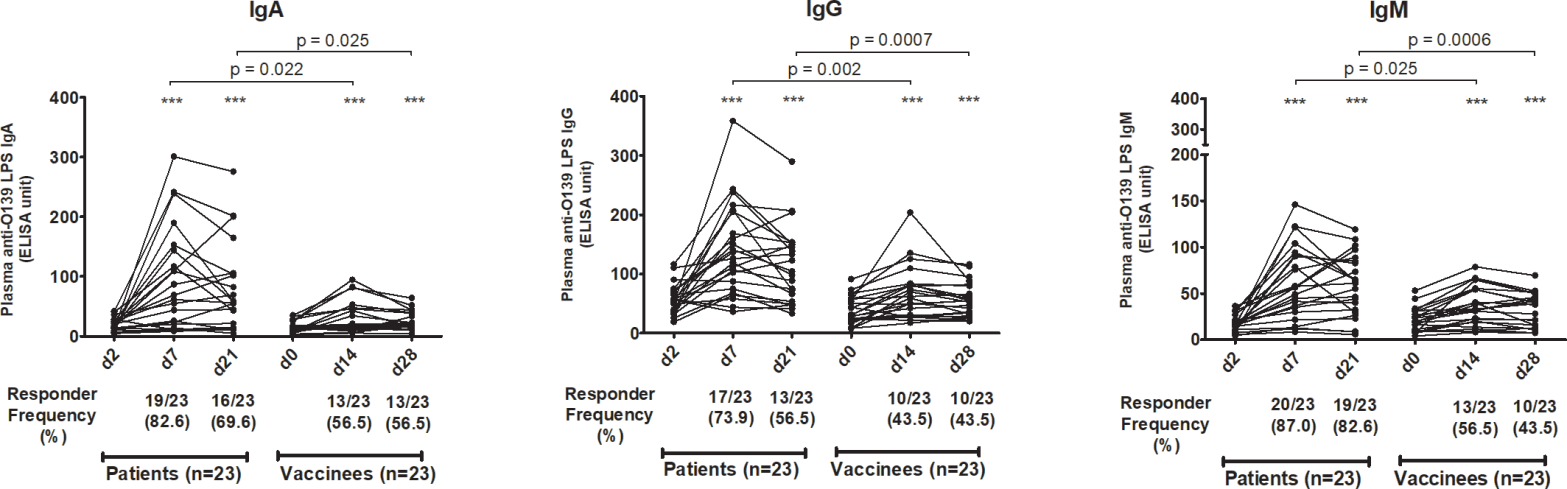
Plasma antibody responses against *V. cholerae* O139 lipopolysaccharide in naturally infected patients and bivalent O1/O139 vaccinees. *P* values represent statistical differences of the mean between patients and vaccinees. Asterisks represent statistically significant differences in immune responses within patients or vaccinees compared to baseline (***P ≤ 0.001, **P ≤ 0.01, and *P ≤ 0.05). Responder frequencies are shown in parentheses below the X-axes (see text).

We assessed antibody responses in plasma or serum against OSP, the terminal tetrasaccharide of OSP, LPS, and capsule of *V. cholerae* O139 in patients and vaccinees across all isotypes and across all three time points. In patients, plasma IgA, IgG, and IgM antibodies targeting OSP (Fig. 1), its terminal tetrasaccharide (Fig. 2), and LPS (Fig. 3) increased significantly at day 7 (early convalescent stage) compared to day 2 (acute stage) and remained significantly elevated at day 21 (late convalescent stage) compared to baseline. Similarly, in vaccinees, plasma IgA, IgG, and IgM antibodies against OSP, tetrasaccharide, and LPS increased significantly at day 14 (14 days after the first dose of vaccine) compared to day 0 and remained significantly elevated at day 28 (14 days after the second vaccination) compared to baseline, except for anti-OSP IgM responses that had returned to baseline on day 28 (Fig. 1 to 3). No significant increases in IgA, IgG, and IgM antibodies against the capsule of *V. cholerae* O139 were observed in patients or vaccinees (Fig. S1).

The magnitude of antibody responses against OSP, the OSP terminal tetrasaccharide, and LPS was significantly higher in patients compared to those in vaccinees in all antibody isotypes and at all follow-up time points (Fig. 1 to 3). Responder frequencies were also significantly higher in patients compared to vaccinees (Fig. 1 to 3).

Significant correlations were observed between anti-OSP and anti-terminal tetrasaccharide antibodies, anti-LPS and anti-OSP antibodies, and between anti-LPS and anti-tetrasaccharide antibodies in both patients and vaccinees (Fig. S2).

### Vibriocidal antibody responses

**Fig 4:**
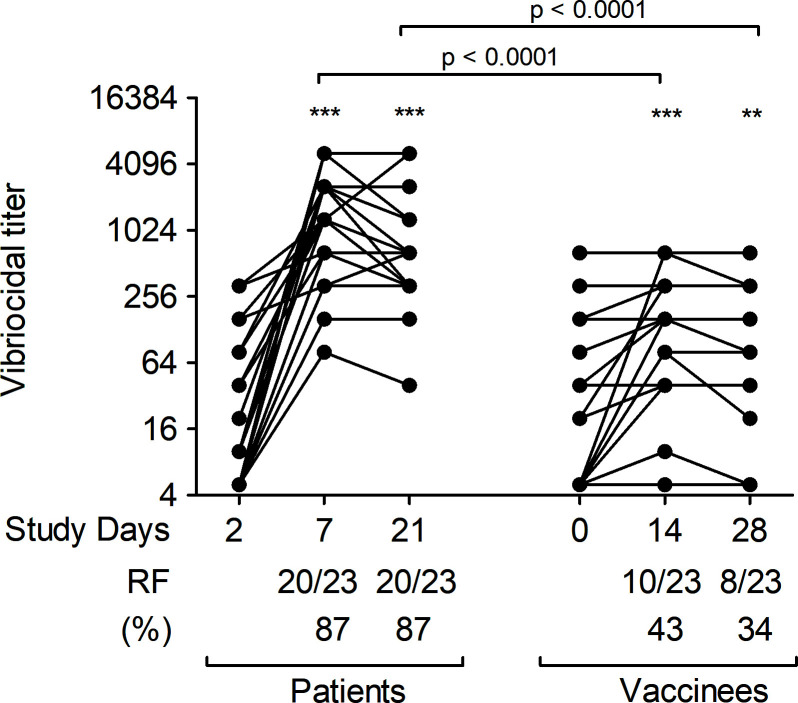
Vibriocidal antibody responses against *V. cholerae* O139 strain CIRS134B (thinly encapsulated) in naturally infected patients and bivalent O1/O139 vaccinees. *P* values represent statistical differences of the mean between patients and vaccinees. Asterisks represent statistically significant differences in vibriocidal titer within patients or vaccinees compared to baseline (***P ≤ 0.001, **P ≤ 0.01, and *P ≤ 0.05). Responder frequency) is defined as the percentage of subjects with a ≥fourfold increase in vibriocidal titer over the baseline.

We assessed vibriocidal antibody titers in plasma against *V. cholerae* O139 in both patients and vaccinees at all time points (Fig. 4). Vibriocidal responses in patients peaked on day 7 compared to day 2 and remained significantly elevated over baseline on day 21. Similarly, vibriocidal responses in vaccinees also increased significantly after the first dose of vaccine compared to baseline. There was no significant boosting of vibriocidal responses after the second dose of vaccine but values remained higher than baseline. Vibriocidal responses were significantly higher in patients than in vaccinees across all follow-up time points, with no differences in responses between groups at baseline. Vibriocidal responder frequency (RF) was also significantly higher in patients compared to vaccinees (Fig. 4).

### Comparison of plasma antibody responses to OSP, terminal tetrasaccharide, and LPS with vibriocidal antibody responses

We compared vibriocidal antibody responses against responses to OSP, terminal tetrasaccharide of OSP, and LPS in both patients and vaccinees (Fig. S2). IgM responses to these antigens are best correlated with vibriocidal responses among isotype responses. IgA and IgG responses to these antigens also significantly correlated with vibriocidal responses in both patients and vaccinees, except for IgA antibody responses against terminal tetrasaccharide in vaccinees (Fig. S2).

### Comparison of plasma antibody responses to OSP related to baseline vibriocidal antibody responses

In order to assess whether previous exposure to *V. cholerae* O139 had an effect on subsequent immune responses, we compared anti-OSP responses within patients (Fig. S3) and within vaccinees (Fig. S4) separately based on baseline vibriocidal titer. No differences in IgA, IgG, and IgM anti-OSP responses were observed at any later time points between study participants with baseline vibriocidal titers ≥80 versus <80.

### Plasma antibody responses to OSP, LPS, and vibriocidal antibody responses of vaccine recipients in young children

**Fig 5:**
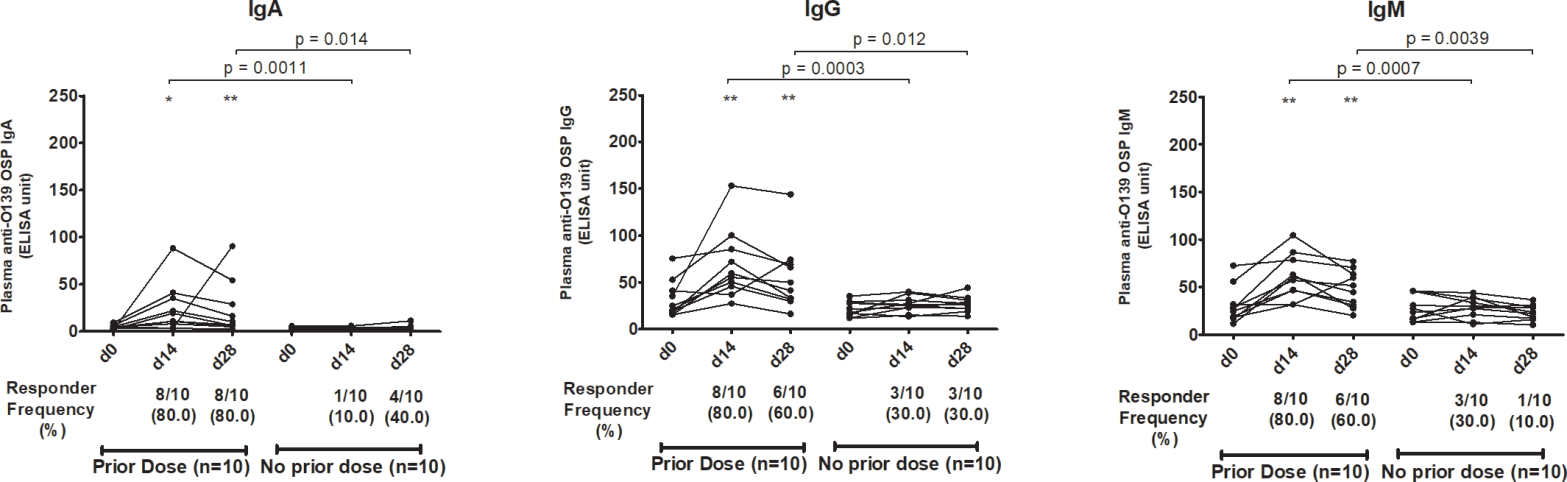
Boosting effect in children vaccinees (<5 yr of age) against *V. cholerae* O139 O-specific polysaccharide. *P* values represent statistical differences of the mean between the two groups of recipients of 2-dose O1/O139 vaccine in 2017: those who received a single dose of O1/O139 vaccine 3 yr previously and those who received a single dose of placebo then. Asterisks represent statistically significant differences in immune responses within the vaccinee group compared to baseline (***P ≤ 0.001, **P ≤ 0.01, and *P ≤ 0.05). Responder frequencies are shown in parentheses below the X-axes (see text).

**Fig 6:**
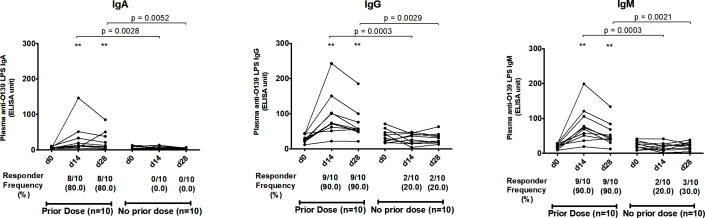
Boosting effect in children (<5 yr of age) receiving a bivalent O1/O139 vaccine against *V. cholerae* O139 lipopolysaccharide). *P* values represent statistical differences of the mean between the two groups of recipients of two-dose O1/O139 vaccine in 2017: those who received a single dose of O1/O139 vaccine 3 yr previously and those who received a single dose of placebo then. Asterisks represent statistically significant differences in immune responses within the vaccinee group compared to baseline (***P ≤ 0.001, **P ≤ 0.01, and *P ≤ 0.05). Responder frequencies are shown in parentheses below the X-axes (see text).

**Fig 7:**
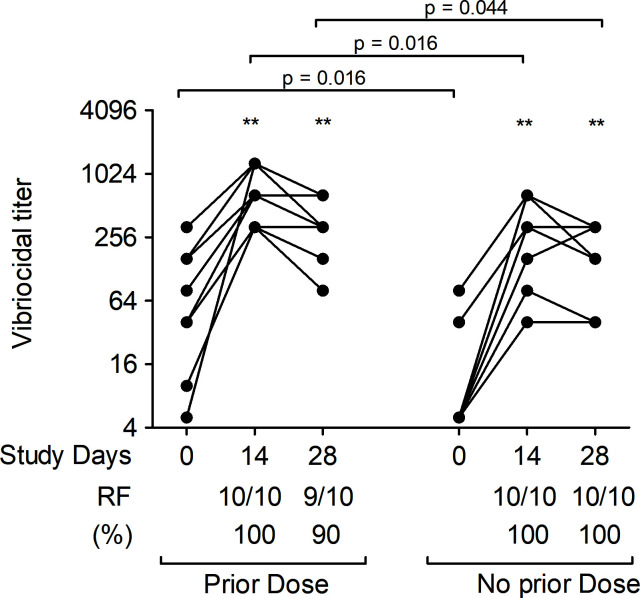
Vibriocidal antibody responses against *V. cholerae* O139 strain CIRS134B (thinly encapsulated) in children (<5 yr of age) receiving a bivalent O1/O139 vaccine. *P* values represent statistical differences of the mean between the two groups of recipients of two-dose O1/O139 vaccine in 2017: those who had received a single dose of O1/O139 vaccine 3 yr previously and those who had received a single dose of placebo then. Asterisks represent statistically significant differences in vibriocidal titer within the group compared to baseline (***P ≤ 0.001, **P ≤ 0.01, and *P ≤ 0.05). Responder frequency is defined as the percentage of participants with a ≥ fourfold increase in titer over the baseline.

In order to further address the potential impact of previous exposure to *V. cholerae* O139, we assessed anti-OSP, anti-LPS, and vibriocidal antibody responses in 20 child vaccinees who were under 5 yr of age in 2017. These individuals were chosen since they had been born after *V. cholerae* O139 was widely circulating in Bangladesh, in order to lessen the possibility of any previous unknown exposure to *V. cholerae* O139. Among these 20 vaccinees, we identified 10 who had received a single dose of OCV in a previous single dose vaccine study performed 3 yr previously in 2014 and 10 who had received only a placebo in that earlier study. Among these young children, only vaccinees who had received a prior single dose of OCV in 2014 mounted significant IgA, IgG, and IgM anti-OSP (Fig. 5) and anti-LPS (Fig. 6) responses against O139 at day 14 compared to day 0, with the responses remaining significantly higher than baseline on day 28. Interestingly, unlike anti-OSP and anti-LPS responses that were only seen in previously vaccinated children, significant vibriocidal responses were observed at day 14 compared to day 0 among both groups of vaccinees (Fig. 7).

## DISCUSSION

Protection against cholera is serogroup-specific, and serogroup-specificity is determined by the O-specific polysaccharide component of bacterial LPS (13). Previous infection with *V. cholerae* O1 does not provide protection against O139 and vice versa (12), despite the fact that *V. cholerae* O139 evolved from *V. cholerae* O1 and expresses identical proteins including cholera toxin (11). The major difference between *V. cholerae* O139 and O1 is in the *rfb* genes involved in OSP synthesis (21, 30, 31) and the presence of a capsule in O139, with the O139 capsule comprised of a polymer of O139 OSP (15, 18 – 21). Specifically, the OSP of *V. cholerae* O1 is comprised of 10–20 repetitive (1→2)-α-linked (4-*N*-3-deoxy-l-*glycero*-tetronyl)-perosamines, with or without a 2-*O*-methyl group on the terminal saccharide determining the Ogawa or Inaba serogroup, respectively (32, 33). In comparison, the OSP of *V. cholerae* O139 is a single hexasaccharide containing *N*-acetyl-d-quinovosamine (QuiNAc), d-galacturonic acid (GalA), *N*-acetyl-d-glucosamine (GlcNAc), d-galactose (Gal), and two colitose (Col) residues. It contains two negatively charged groups: a carboxyl group of d-galacturonic acid and a cyclic phosphate bound to *O*-4 and *O*-6 of d-galactose (18, 19, 34). The capsule of *V. cholerae* O139 contains a flexible, complex, and branched polymer of this hexasaccharide (18, 19, 34).

In our analysis, we found that patients with cholera caused by *V. cholerae* O139 develop prominent OSP-specific immune responses, despite the presence of a capsule. These immune responses mirrored immune responses against LPS and were largely directed against the terminal tetrasaccharide component of the O139 non-repeating OSP hexasaccharide. We have previously shown that the tetrasaccharide is the most immunogenic component of the *V. cholerae* O139 OSP (22), suggesting pocket-like interaction between antibody and antigen, as observed for *V. cholerae* O1 OSP (33) and other bacterial surface polysaccharides (35 – 38).

In our previous analysis profiling immune responses against a range of O139-related saccharides, we performed an analysis using pooled plasma samples (*n* = 10) collected on day 2 and day 7 from patients with cholera caused by *V. cholerae* O139 in Bangladesh and pooled samples (*n* = 10; enrollment and 7 d after receipt of vaccine dose one and 7 d after vaccine dose two) from Haitian recipients of bivalent OCV (22). Here, we report a detailed analysis at the individual patient/participant level of patients and vaccinees in Bangladesh, an area in which *V. cholerae* O139 had previously emerged. In our prior analysis of immune responses in Haitian vaccinees, we found only low-level IgM OSP-specific responses and no induction of IgA or IgG responses (22). In contrast, in our current analysis of immune responses in the age-gender-blood group matched vaccinees compared to naturally infected patients in Bangladesh, we found significant IgA, IgG, and IgM OSP-specific responses following vaccination, although these responses were less prominent than those induced following natural infection. It is possible that these higher immune responses following natural disease may reflect in part the impact of cholera toxin, a potent immunoadjuvant, during natural disease; the bivalent vaccine does not contain this immunoadjuvant. We considered that these different levels of immunogenicity in Bangladeshi vaccinees versus Haitian vaccinees may reflect different previous exposure to *V. cholerae* O139 in the two populations. *V. cholerae* O139 has never been reported in Haiti. In comparison, *V. cholerae* O139 caused large outbreaks for over a decade in the 1990s (2) and then smaller outbreaks in the early 2000s (39) in Bangladesh and is still occasionally identified in stool cultures (6, 8) and in the environment in Bangladesh (40, 41).

We were constrained in our current analysis in that available samples of serum of our naturally infected patients from 2002 were largely from adults (20 of 23 samples). Since we matched to this cohort, our vaccinee samples were, therefore, also largely from adults. The naturally infected samples had been collected in 2002, years before the vaccine study in 2017, since O139 had disappeared as a significant cause of cholera when the bivalent OCV was developed and deployed. As such, the adults included in our vaccine cohort were children when Bangladesh was afflicted by large outbreaks of cholera caused by O139, and our detection of O139-specific immune responses in our vaccinees could possibly represent a boosting response from previous undocumented exposure. To address this possibility, we first analyzed O139-specific OSP responses in Bangladeshi vaccinees based on a baseline vibriocidal titer of 80 or greater (possibly consistent with previous exposure). However, we were unable to discern significant differences in subsequent OSP responses by this cohort analysis, perhaps because of the poor predictive value of the vibriocidal parameter in O139 (42, 43) and its unknown predictive value to identify a possible exposure 10–20 yr prior.

We, therefore, performed an additional analysis using vaccinees who were children born after *V. cholerae* O139 had receded in Bangladesh. In this sub-analysis, we had samples from children who received the standard two doses of bivalent vaccine (day 0 and 14) in 2017 and who had been randomized to receive either a single dose of bivalent OCV or a placebo in 2014. We found no significant induction of immune responses against OSP in immunologically naïve Bangladeshi children (vaccine recipients in 2017 who were born after O139 cholera had receded in Bangladesh and who had received a placebo in the previous single-dose vaccine study in 2014). However, we detected significant responses in children who had received a prior single dose of bivalent (O1/O139) vaccine 3 yr previously. These results are consistent with the poor O139 OSP-specific immunogenicity of initial vaccination with bivalent OCV that we observed in immunologically naïve Haitian vaccinees (22) but also suggest that such vaccination can prime OSP and related O139-specific responses that can subsequently be boosted. As such, previous exposure or vaccination would suggest induction of memory responses.

We found a high correlation of OSP-hexasaccharide, OSP-terminal tetrasaccharide, LPS, and vibriocidal responses following natural infection across antibody isotypes (IgA, IgM, and IgG). Vibriocidal responses during *V. cholerae* O139 can vary depending on the strain, capsule thickness, growth media, inoculum size, and concentration of complement (44). To maximize the detection of vibriocidal responses, we used a thinly encapsulated *V. cholerae* O139 strain (22, 24). Although we found a correlation between OSP responses and vibriocidal responses in vaccinees, the degree of these correlations was less prominent than those observed following natural disease. Specifically, a number of vaccinees had moderate vibriocidal responses in the setting of low OSP-specific responses. We have previously shown that the affinity of *V. cholerae* O1 OSP-specific antibodies does not predict functionality, including vibriocidal activity (45); specifically, low-affinity OSP-specific antibodies can have significant vibriocidal activity, and our current observations may reflect this effect. Another possibility is that these responses could reflect the presence of antibodies to antigens other than OSP. We also found that IgA antibodies that targeted terminal tetrasaccharide in vaccinees did not correlate with vibriocidal responses. This could be attributed to the fact that the vibriocidal response is a surrogate marker of protection against cholera and assesses complement-mediated bacterial lysis *in vitro* (43), while IgA does not bind complement via the classical pathway.

*V. cholerae* O139 is encapsulated, and the capsule is comprised of a polymer of the *V. cholerae* O139 OSP hexasaccharide (15, 18 – 21). Despite our ability to detect antibody responses to the O139 OSP hexasaccharide, we were unable to detect significant increases in antibody responses to the capsule in both patients and vaccinees, perhaps suggesting altered immunologic display of the polysaccharide in its polymerized form, and perhaps underscoring that the ability of anti-OSP and anti-LPS antibodies to provide mechanistic protection against O139 cholera is yet to be determined.

In summary, naturally acquired cholera caused by *V. cholerae* O139 in an endemic area induces prominent OSP-specific responses despite capsule. These responses correlate with LPS and vibriocidal responses and are primarily directed against the terminal tetrasaccharide of OSP. In comparison, the bivalent OCV does not induce detectable OSP responses in immunologically naïve individuals but boosts OSP-specific responses in individuals previously exposed (or primed) to *V. cholerae* O139 antigens via community exposure or vaccination. The degree of protection afforded by such priming is unknown. Indeed, the ability of bivalent OCV to protect against cholera caused by *V. cholerae* O139 has never been demonstrated, and efficacy can no longer be easily assessed since the incidence of *V. cholerae* O139 infection is now rare; assessing protection afforded by vaccination would now require use of a human challenge model. Moreover, since O139 has not been of public health importance for many years, new simplified versions of OCV are being developed that do not contain *V. cholerae* O139 (46). It is unknown why *V. cholerae* O139 has receded but should it re-emerge, our data suggest that oral ingestion of killed O139 can prime immune responses. However, it remains unclear whether such priming would protect against virulent *V. cholerae* O139.

## Data Availability

The raw data supporting the findings of this article will be made fully available by the authors, without undue reservation.

## SUPPLEMENTAL MATERIAL

Fig. S1 msphere.00255-23-s0001.tif Plasma antibody responses against *Vibrio cholerae* O139 capsule in naturally infected patients, and bivalent O1/O139 vaccinees.

Fig. S2 msphere.00255-23-s0002.tif Correlation heatmap showing pairwise spearman correlation matrices of *V. cholerae* O139 antigen-specific antibody responses for patients (left) and bivalent vaccinees (right).

Fig. S3 msphere.00255-23-s0003.tif Differences in plasma antibody responses against *Vibrio cholerae* O139 O-specific polysaccharide (OSP) in naturally infected patients based on vibriocidal titer at "baseline" (day 2 post-infection).

Fig. S4 msphere.00255-23-s0004.tif Differences in plasma antibody responses against *Vibrio cholerae* O139 O-specific polysaccharide (OSP) in vaccinees based on vibriocidal titer on the day of first dose of vaccination.

ASM does not own the copyrights to Supplemental Material that may be linked to, or accessed through, an article. The authors have granted ASM a non-exclusive, world-wide license to publish the Supplemental Material files. Please contact the corresponding author directly for reuse.
