# Supplementary figures and images for: Comparison of O-specific polysaccharide responses in patients following infection with Vibrio cholerae O139 versus vaccination with a bivalent (O1/O139) oral killed cholera vaccine in Bangladesh
Source: mSphere. 2023 Aug 30;8(5):e00255-23. doi: 10.1128/msphere.00255-23 (PMC10597347; doi:10.1128/msphere.00255-23)

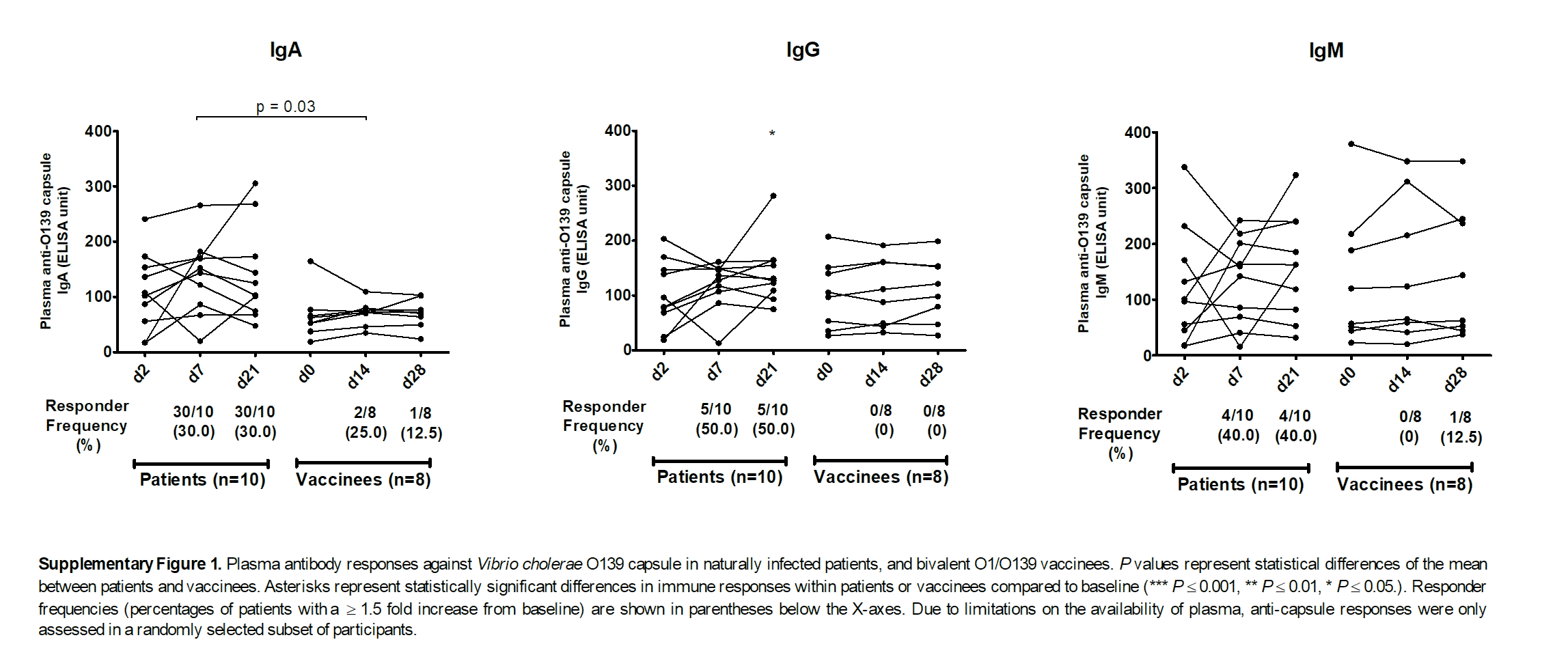

Supplement: Fig. S1 — Plasma antibody responses against Vibrio cholerae O139 capsule in naturally infected patients, and bivalent O1/O139 vaccinees. [file msphere.00255-23-s0001.tif]

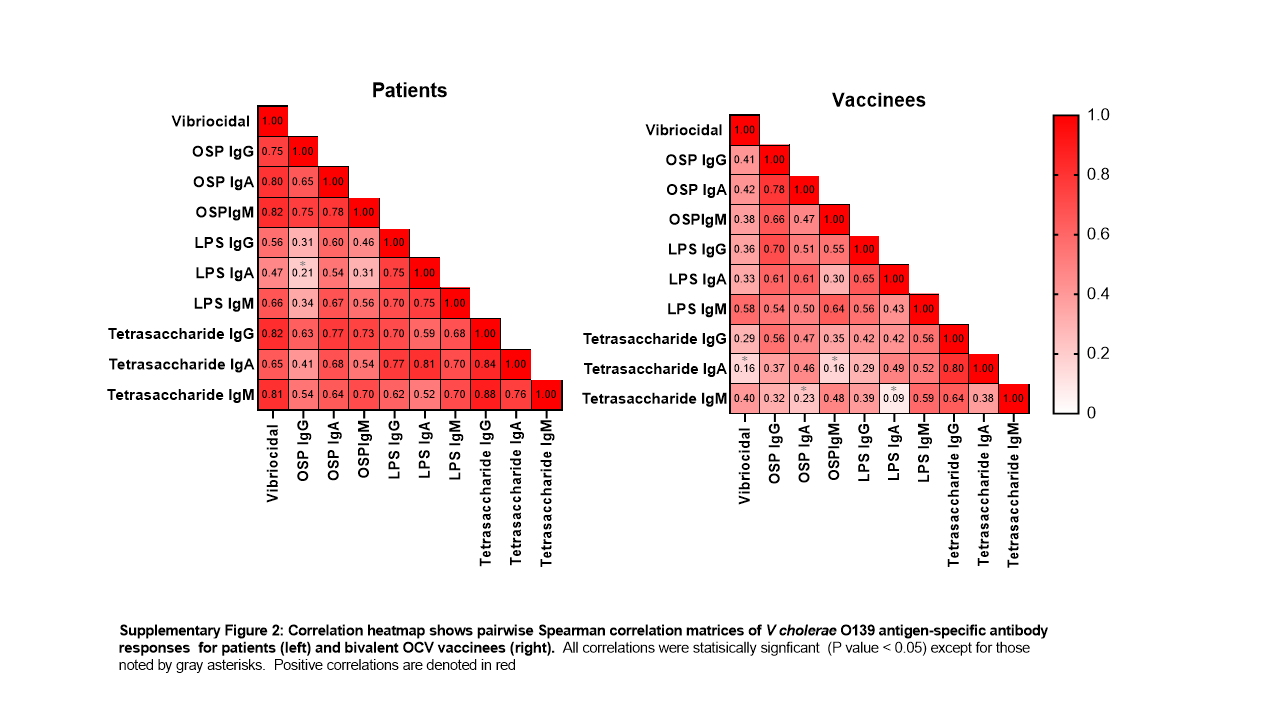

Supplement: Fig. S2 — Correlation heatmap showing pairwise spearman correlation matrices of V. cholerae O139 antigen-specific antibody responses for patients (left) and bivalent vaccinees (right). [file msphere.00255-23-s0002.tif]

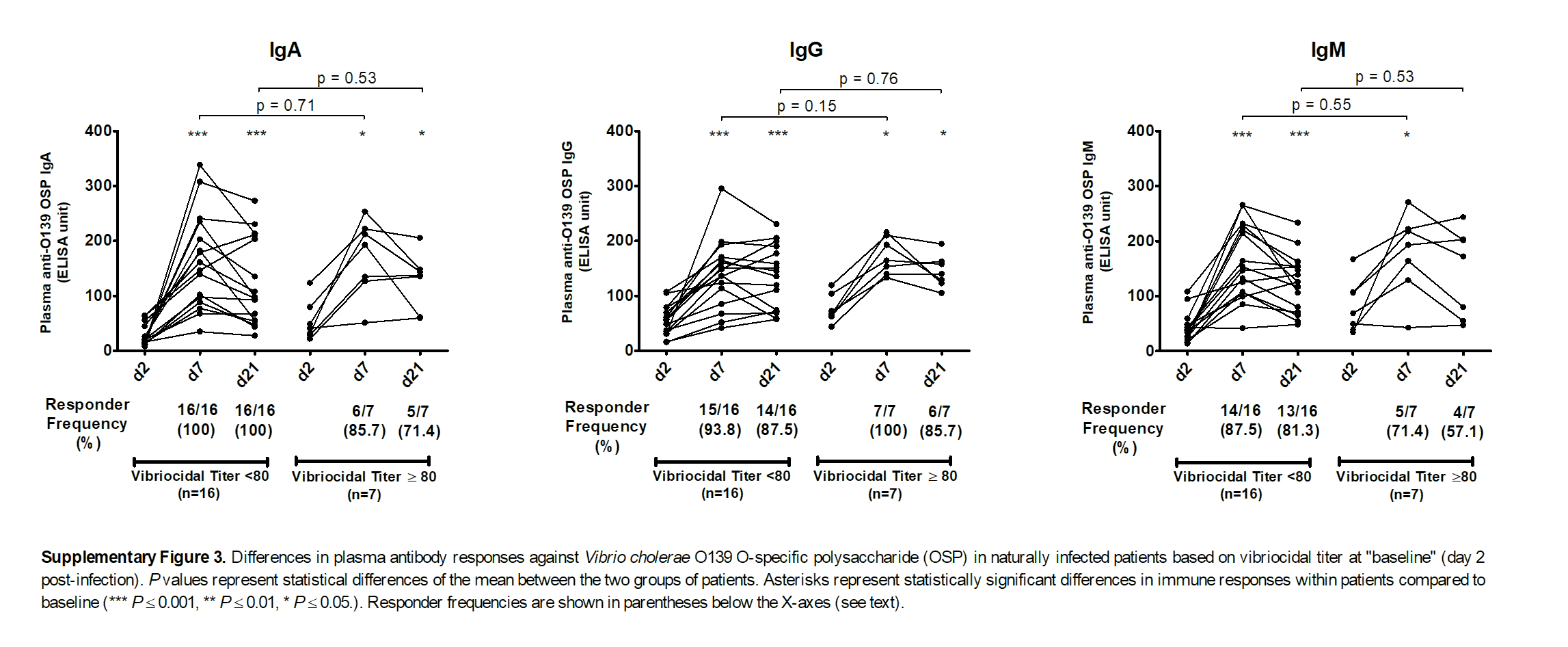

Supplement: Fig. S3 — Differences in plasma antibody responses against Vibrio cholerae O139 O-specific polysaccharide (OSP) in naturally infected patients based on vibriocidal titer at "baseline" (day 2 post-infection). [file msphere.00255-23-s0003.tif]

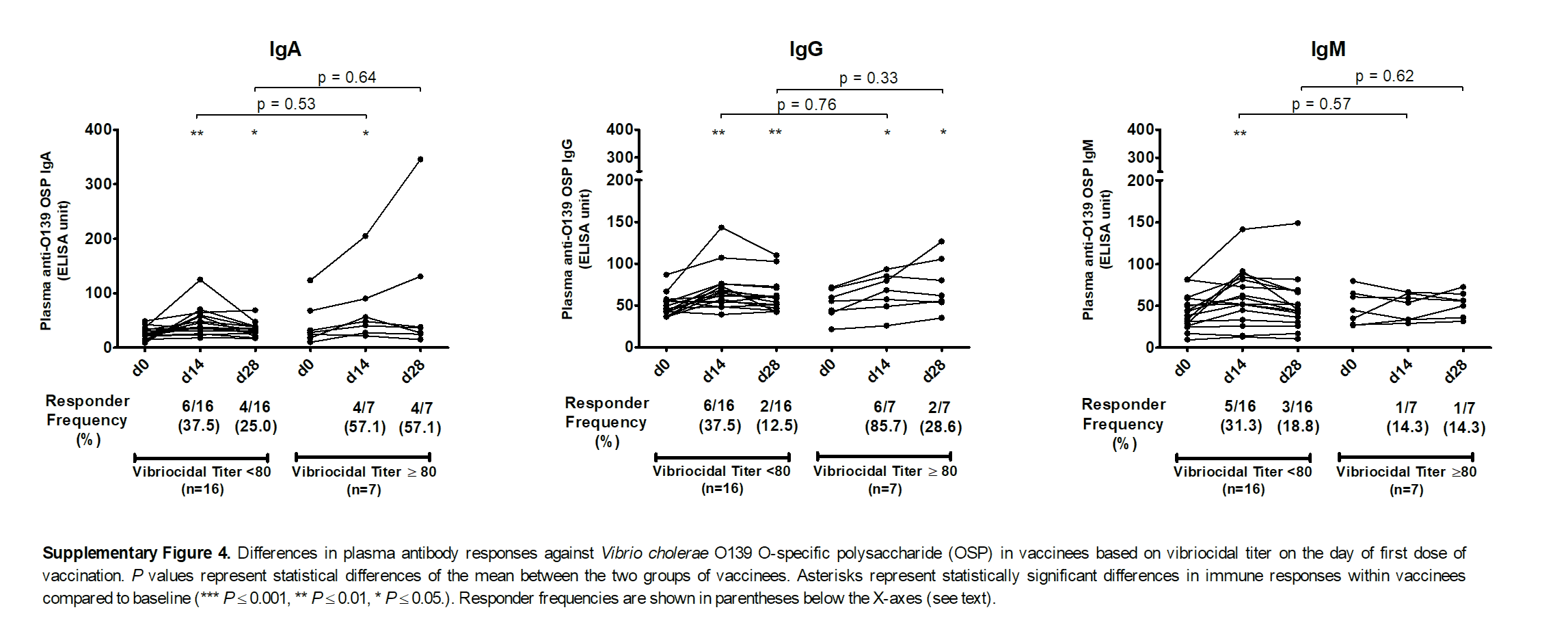

Supplement: Fig. S4 — Differences in plasma antibody responses against Vibrio cholerae O139 O-specific polysaccharide (OSP) in vaccinees based on vibriocidal titer on the day of first dose of vaccination. [file msphere.00255-23-s0004.tif]
